# A directionally selective collision-sensing visual neural network based on fractional-order differential operator

**DOI:** 10.3389/fnbot.2023.1149675

**Published:** 2023-04-21

**Authors:** Yusi Wang, Haiyang Li, Yi Zheng, Jigen Peng

**Affiliations:** Machine Life and Intelligence Research Centre, School of Mathematics and Information Science, Guangzhou University, Guangzhou, China

**Keywords:** direction selectivity, fractional-order differential operator, neural modeling, collision sensing, real complex scene

## Abstract

In this paper, we propose a directionally selective fractional-order lobular giant motion detector (LGMD) visual neural network. Unlike most collision-sensing network models based on LGMDs, our model can not only sense collision threats but also obtain the motion direction of the collision object. Firstly, this paper simulates the membrane potential response of neurons using the fractional-order differential operator to generate reliable collision response spikes. Then, a new correlation mechanism is proposed to obtain the motion direction of objects. Specifically, this paper performs correlation operation on the signals extracted from two pixels, utilizing the temporal delay of the signals to obtain their position relationship. In this way, the response characteristics of direction-selective neurons can be characterized. Finally, ON/OFF visual channels are introduced to encode increases and decreases in brightness, respectively, thereby modeling the bipolar response of special neurons. Extensive experimental results show that the proposed visual neural system conforms to the response characteristics of biological LGMD and direction-selective neurons, and that the performance of the system is stable and reliable.

## 1. Introduction

With the rapid development of science and technology, intelligent mobile machines have come to have increasingly important roles in people's lives. A variety of intelligent mobile machines, including robots, unmanned aerial vehicles, ground vehicles, and automatic patrol cars, make various aspects of our lives more technical, automatic, and intelligent. It is a great challenge to endow such machines with the capacity to respond to the dynamic visual world in real-time; however, this is of great significance in their development (Cizek and Faigl, 2019; Yulia et al., 2020). Among many visual functionalities, sensing the threat of collision with external moving objects (collision sensing for short) is one of the most important abilities for intelligent mobile machines and is an important guarantee of safe driving by intelligent machines (Dietmueller et al., 2017; Hartbauer, 2017). Therefore, it is particularly important to develop efficient, reliable, and fast collision-sensing vision systems.

Spike neural networks are an important research topic in the field of brain-inspired intelligence. These networks process information by simulating the pulse signal transmission between neurons, which results in higher energy efficiency and faster response times, making them advantageous for applications such as autonomous driving and robotics. In recent years, research on Spike neural networks has rapidly developed and achieved fruitful results (Samanwoy and Hojjat, 2009; Bing et al., 2018; Neftci et al., 2019; Tavanaei et al., 2019; Fang et al., 2021a,b; Shalumov et al., 2021; Yang et al., 2022). However, Spike neural networks still have the following shortcomings: (1) The neuron model is more complex. The neuron model of Spike neural networks is more complex than traditional neuron models, requiring more computing resources and higher computational accuracy. (2) Network structure design is more difficult. The network structure design of Spike neural networks requires more specialized knowledge and experience, making it more challenging to design. Therefore, we need to develop a network system that is structurally simple and efficient.

Insects' visual system has a relatively simple structure, with fewer neurons inside the system. It perceive changes in information on the retina and make a series of responses through neuron transmission, serving as a bridge connecting the brain and the visual world. However, even the simple vision systems of insects can reliably guarantee several behaviors including collision sensing, collision avoidance, fast target detection, and tracking during flight, especially efficient motion adaptation in complex dynamic scenes (Rind and Simmons, 1999; Rind et al., 2003; Santer et al., 2005; Yue and Rind, 2006). For example, locusts rely on their advanced vision systems to fly hundreds of miles during migration without colliding with each other, and their collision avoidance rate in dense forests during foraging is as high as 98% (Kennedy, 1951; Thorson, 1966). In this work, considering the cost, power consumption, and reliability of intelligent systems, we take the insect vision system as a starting point to study and simulate a working mechanism with the aim of developing a simple, efficient, and reliable collision sensing system.

The insect visual nervous system includes special neurons called lobular giant motion detectors (LGMDs), which show strong neural responses to the looming motion of objects, whereas they hardly respond to objects far away (O'Shea and Williams, 1974; Rind and Bramwell, 1996). More precisely, LGMDs' dendritic arborizations ramify in the third neuropil (lobula) of locust optic lobes and consist of three dendritic subfields. The main subfield is thought to receive an excitatory retinotopic projection that is sensitive to motion (Rowell et al., 1977; Sztarker and Rind, 2014). Neurophysiological studies have shown that LGMD form part of the fast neural pathway in the early collision warning systems of insects, and that their purpose is to trigger escape or avoidance behaviors when an insect encounters danger from a looming object (Rind and Simmons, 1992; Judge and Rind, 1997).

In the past few years, many advances have been made in biological research on LGMD neurons and their afferent pathways, and a series of collision-sensing neural networks based on these advances have been proposed. Such models are based on two main types of neural response of LGMDs to looming objects. (1) During the looming process of objects, the rate at which spikes are emitted by neurons increases continuously (O'Shea and Williams, 1974; Judge and Rind, 1997). (2) During the looming process of objects, the spike rate of neurons reaches a peak at or before the collision, which depends only on the image size of the looming object on the retina (Hatsopoulos et al., 1995; Gabbiani et al., 1999). However, existing collision-sensing network models are only used to detect the occurrence of a collision; they cannot obtain additional motion information about the collision objects, such as the motion direction, motion speed, or collision location. Therefore, further study of the motion of collision objects is needed to enhance the function of collision-sensing visual neural networks.

Neurophysiological studies have identified special visual neurons in insects with a preference for specific directional motion information, called directionally selective neurons (Barlow and Hill, 1963; Stavenga and Hardie, 1989). They respond strongly to motion oriented along a preferred direction but show weak or no response, or even a fully opponent response, to null-direction motion. The null direction is 180° from the preferred direction. Directionally selective neurons can quickly and reliably extract visual motion information in different directions and show a strong response to the motion in the preferred direction. At present, the postsynaptic pathways of directional selective neurons are still under investigation, but it is obvious that identifying the direction of a collision object's movement enables insects to choose the correct collision avoidance route, which is an indispensable advantage in collision escape or avoidance tasks.

To date, there has been relatively little research systematically modeling the motion direction of collision objects. In a pioneering study based on the classic LGMD model, Yue and Rind (2013) made the inhibitory waves of neurons propagate in a specific direction through delaying the time step and weakened the responses of neurons in a specific direction, thereby extracting motion information in a specific direction. Zhang et al. (2015) introduced the Reichardt detector model into an artificial network system to determine the direction of moving objects. Fu and Yue (2017) used a delay filter based on an ON-OFF visual path to extract information in four directions in horizontal and vertical positions in space. Huang et al. (2022) constructed a directionally selective visual processing module based on a spatio-temporal energy framework and extracted motion information for eight directions in space through Gabor filtering. Although some progress regarding directional selectivity was made in these studies, existing models still have a number of shortcomings. (1) The existing models all use the first response mode of LGMDs to study directional selectivity; there has not yet been any attempt to systematically model and analyze directionally selective neurons based on the second response mode using strict mathematical methods. Based on the first response mode of LGMD, Yue and Rind (2013); Zhang et al. (2015), and Fu and Yue (2017) used different methods to depict the directional selectivity of neurons, but these methods all relied on the use of a threshold to make judgments in practical applications. However, it is often difficult to choose an appropriate threshold in different scenarios. (2) The work of Fu and Yue (2017) and Huang et al. (2022) mainly relied on the use of filters to extract direction information; they did not systematically study the selective response of neurons in the preferred direction. In addition, the spatiotemporal energy based model is considered to be mathematically equivalent to the Reichardt-type model. When its temporal and spatial filter settings are the same, it can achieve similar specific functional characteristics. (3) Existing models use the first-order differential operator or difference network framework to model directionally selective neurons. However, a large number of biological studies have shown that the neural dynamics followed by the neuron response should be of fractional order, and a fractional-order differential operator is required to accurately describe the biological logic of the brain neural response process (Podlubny, 2013; Teka et al., 2013; Chen et al., 2018; Wan and Jian, 2019). Therefore, further systematic studies using strict mathematical methods are required to model the directional selectivity in LGMD-based collision-sensing neural networks and elucidate their internal computing principles.

In this paper, a directionally selective fractional-order LGMD collision-sensing visual neural network, called DFLGMD, is proposed. As well as sensing the looming motion of a collision object, this model can obtain the direction of the looming object. Moreover, DFLGMD is based on the second response mode of LGMD neurons; it does not rely on a threshold to make judgments in practical applications. In the proposed neural network model, a new correlation mechanism is used to correlate the signals obtained from two pixels with directional information, so as to characterize the special selective response of directionally selective neurons to movements in the preferred direction. Then, an ON-OFF visual channel is introduced to act on the correlation output. The purpose is to divide the motion signal into parallel channels, code the increases and decreases in brightness, and thus model the bipolar response of special neurons. Finally, a direction-selective neuron is fused with the fractional-order collision-sensing visual neural network, such that the system can obtain the motion direction of the collision object while sensing the collision threat, and further make the correct collision avoidance decision. The proposed visual neural network is systematically studied and tested in different environments. The results show that the DFLGMD visual neural network not only produces reliable response spikes to looming objects but can also accurately obtain the moving direction of looming objects; moreover, its network reliability is better than those of previous methods.

The main contributions of this paper can be summarized as follows.

(1) We develop a new DFLGMD collision-sensing visual neural network and provide a unified and rigorous mathematical description.

(2) An ON-OFF visual channel is introduced into the fractional-order LGMD visual neural network, enabling selectivity of the system for looming bright and dark objects by encoding increases and decreases in brightness, respectively.

(3) The proposed DFLGMD neural network produces a high-fitness and reliable response spike to a looming object, and also accurately identifies the direction of movement of the object.

The remainder of this paper is organized as follows. Section 2 reviews relevant previous work. In Section 3, the proposed new DFLGMD collision-sensing visual neural network is described in detail. In Section 4, the performance of the proposed neural network is verified through experiments, and the results are discussed. We give further discussions in Section 5. In the final section, we conclude this paper.

## 2. Related work

In this section, we review some past work on relevant topics including ON-OFF visual channels, LGMD neurons, and directionally selective neurons. These entities are all found in insects visual systems and have been extensively studied.

### 2.1. ON-OFF visual channel

In an insect's visual system, the retina receives visual signals and then encodes the visual information contained in the signals into sequences of neural responses, which are transmitted to the brain. Different signal transmission pathways process different types of visual information: the cone pathway is involved in encoding bright visual information with relatively high light intensity, and the rod pathway is involved in encoding dark visual information with relatively low light intensity. Under different light adaptation conditions, light enhancement and light attenuation signals are processed through different cone and rod signal transmission pathways, respectively. We call the pathway that transmits light enhancement signals the ON pathway and the pathway that transmits light attenuation signals the OFF pathway.

ON-OFF pathways functioning as information-encoding methods have been discovered in the preliminary visual systems of many animal species (Borst and Euler, 2011; Borst and Helmstaedter, 2015), including flies, mice, and rabbits. Such pathways divide the received visual information into parallel ON or OFF channels and then code the brightness increment (ON) and brightness decrement (OFF) for parallel processing. The response is not sensitive to the spatial change in brightness, but it is sensitive to the temporal change; that is, when the brightness in the circular area of the receptive field changes with time, the output of the visual channel increases or decreases. This property of sensitivity to the change in local brightness with time is the basis of object motion analysis in computer vision.

### 2.2. LGMD neurons

**Figure 1:**
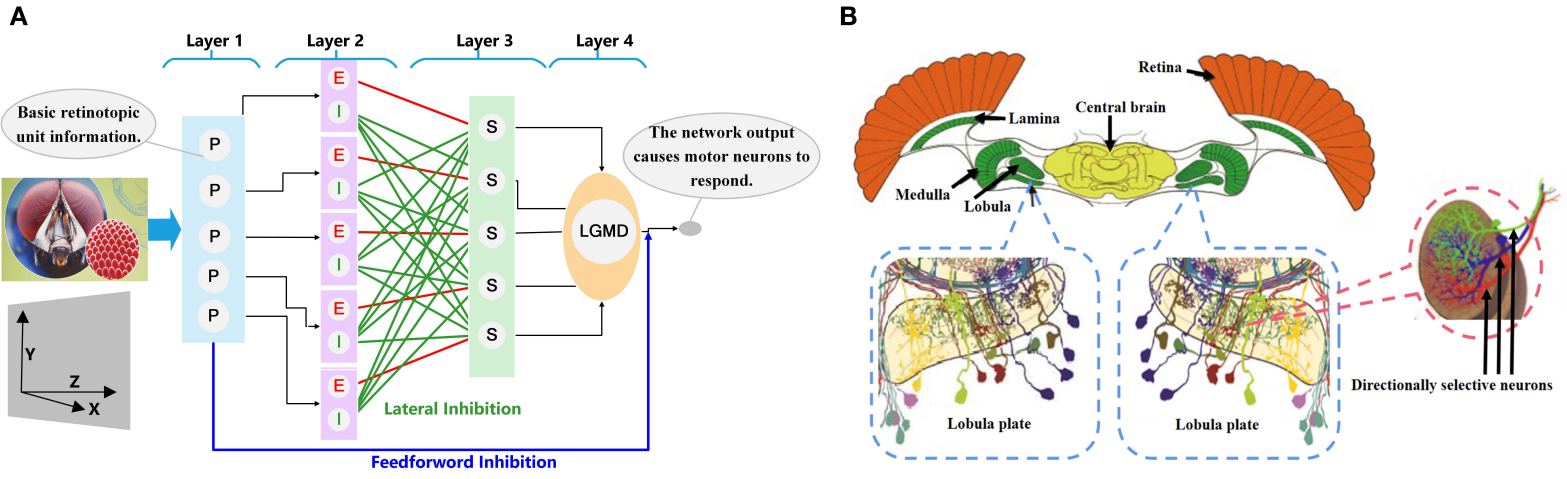
The insect neural system. **(A)** Neural network of LGMD neurons. The neural network is composed of four retinotopically organized layers. **(B)** The biological vision system of insects [modified from Haag and Borst (2002) and Borst et al. (2020)]. The directionally selective neurons are located on the lobular plate, and sense the direction information of movement.

LGMDs are collision-sensitive neurons found in insects' vision systems. They are extremely sensitive and respond strongly to objects looming near the insect on a direct collision course, while exhibiting little or no response to receding objects (O'Shea and Williams, 1974; Rind and Bramwell, 1996). LGMDs are huge neurons. Their dendritic arborizations ramify in the third neuropil (lobula) of insect optic lobes and consist of three dendritic subfields; the main subfield is thought to receive an excitatory retinotopic projection, which is sensitive to motion. An LGMD and descending contralateral motion detector (DCMD) together form a neural circuit (Rowell et al., 1977; Schlotterer, 1977). The fast conducting axon of the neuron is the largest axon in the contralateral nerve cord, which maps to the chest motion center involved in generating jumping and flying movements. The connections between LGMDs and DCMDs are very strong and reliable. Each action potential of an LGMD will trigger an action potential in the corresponding DCMD. On the contrary, under visual stimulation, each action potential in the DCMD is caused by the LGMD (O'Shea et al., 1974; Judge and Rind, 1997). In the whole neural circuit, the LGMD sends out a strong response to the looming object motion and transmits information to the post-synaptic target DCMD neuron, thereby inducing escape behavior. Figure 1 shows the physiological anatomy of an LGMD neuron structure.

The neuronal characteristics of LGMDs have attracted considerable research attention. Building a response model for LGMD neurons not only enables a deeper understanding of biological vision processing but will also help us to develop an efficient, stable, and reliable collision perception vision system for intelligent mobile machines.

### 2.3. Directionally selective neurons

A special directionally selective interneuron is found in the lobular plate of the optic lobe of diptera insects (Figure 1). As a special visual neuron that has a preference for motion information in a specific direction, it can extract motion and direction information about the object from the visual scene projected onto the retina, and quickly and reliably evokes a strong response to information in the preferred direction, while responding weakly or not at all to information in the zero direction (non-preferred direction). Although it has been shown that the direction of object motion is encoded in the two synapses of the lobular lamina (DeSouza and Kak, 2002), because the visual system of insects has a highly organized layered structure and is close to the inner surface of the retina, the relationship between the directionally selective neuron and the visual cortex is unclear (Franceschini, 2004).

Research has shown that directionally selective neurons can be used to extract visual cues for translational motion (Rind and Yue, 2006) and organized with a special structure for collision detection (Yue and Rind, 2007). It is unclear whether these directionally selective neurons can be combined only with this special structure for collision detection. However, results suggest that directionally selective neurons can be effectively and reliably organized for collision sensing, which indicates that similar organizational structures may exist in biological vision systems. This provides strong support for research on collision sensing based on biological vision.

## 3. Model of the directionally selective fractional-order visual neural network

**Figure 2:**
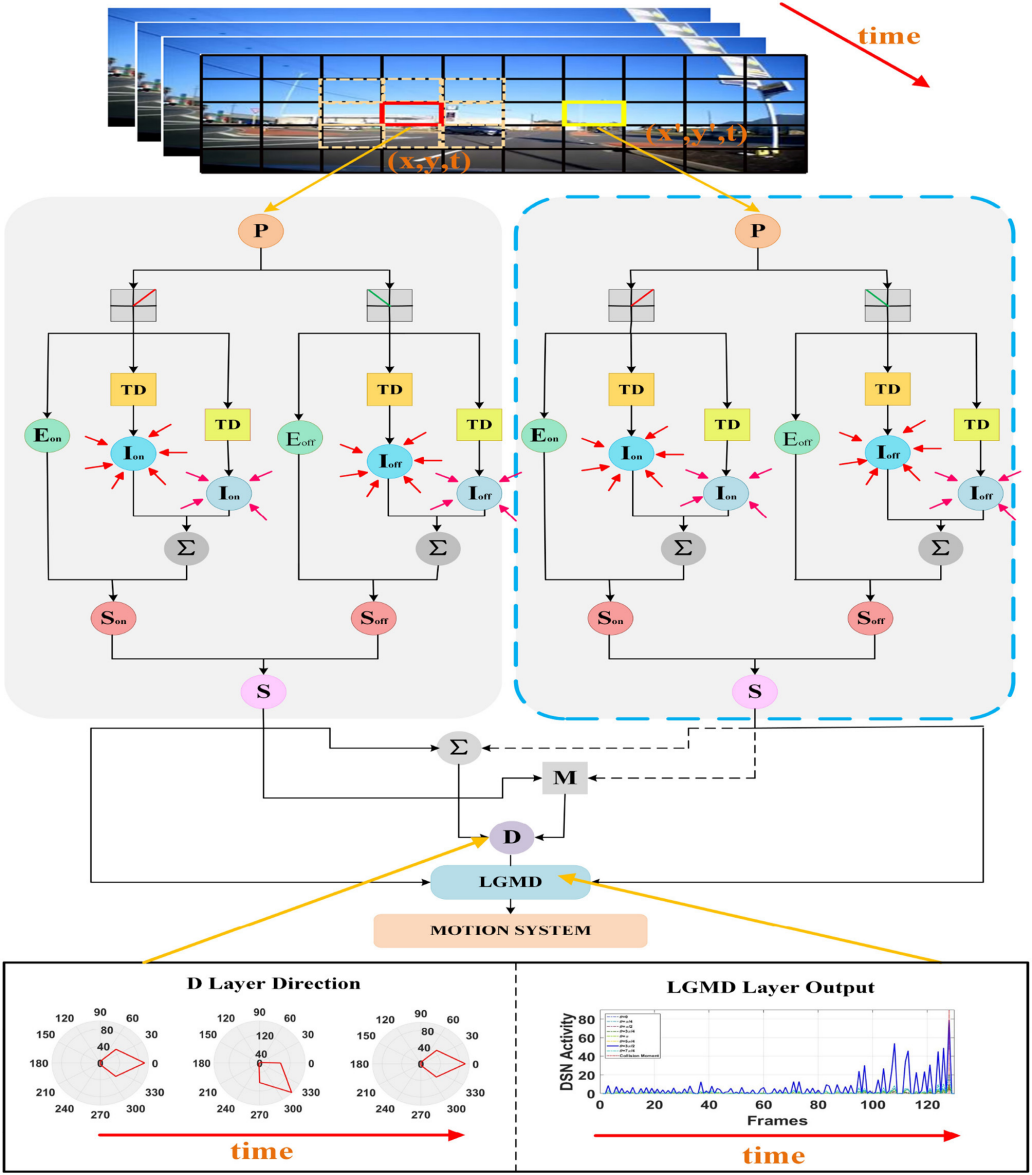
Schematic illustration of the proposed DFLGMD neural network. DFLGMD neural network is composed of photoreceptor, ON/OFF channel, excitability, inhibition, summing cells, directionally selective neuron, and single-cell LGMD cells. For clear illustration, only two photoreceptors at different positions and corresponding downstream processing are described.

In this section, we introduce the neural network model and structure of the proposed DFLGMD. Compared with existing LGMD collision-sensing systems, a key function of DFLGMD is that as well as perceiving collision threats involving moving objects, it can determine the direction of movement of the objects, thereby obtaining more collision information. The neural network is composed of five neural layers, each of which has a special function and cooperates with the others to obtain collision information about moving objects quickly and accurately. Figure 2 shows a schematic diagram of DFLGMD's visual neural network structure. In the following subsections, we introduce the composition and functions of each layer in detail.

### 3.1. Photoreceptor layer

In the insect visual system, the retina can sense an external light stimulus and provide the corresponding external perception information. The retina contains many ommatidia, each of which is composed of eight photoreceptors. Each photoreceptor can observe a small area of the entire visual field. Such multiple-ommatidia visual areas constitute the entire visual field of the retina.

In the DFLGMD neural network, a photoreceptor layer composed of multiple photoreceptors is used as the input layer for the whole network to obtain the brightness information of the entire visual field *L*, that is, to capture the brightness values of changes in the external visual field. The photoreceptor layer directly processes the input original image sequence. Specifically, we use the state variable *P*_*ij*_(*t*) to describe the membrane potential of photoreceptors at position (*i, j*) and time *t*. The dynamics are controlled by a fractional-order differential equation. The model is as follows:


(1) $$\begin{array}{l} \begin{array}{c} D_{t}^{\alpha_{1}}P_{ij}\left(t\right)=g_{leak}\left(V_{rest}-P_{ij}\left(t\right)\right)\\ +\lambda_{ex}L_{ij}\left(t\right)\left(E_{ex}-P_{ij}\left(t\right)\right)+\lambda_{in}L_{ij}\left(t-1\right)\left(E_{in}-P_{ij}\left(t\right)\right), \end{array} \end{array}$$

where $D_{t}^{\alpha_{1}}P_{ij}\left(t\right)$ represents the α_1_-order differential of *P*_*ij*_ with respect to time *t*; *g*_*leak*_ is the leakage conductance, which describes the total passive ion flow through the cell membrane; *V*_*rest*_ is the resting potential of the cell; *E*_*ex*_ and *E*_*in*_ are the excitatory and inhibitory synaptic batteries, respectively, which confine the cell's dynamic range to *E*_*in*_ ≤ *P*_*ij*_ ≤ *E*_*ex*_; and λ_*ex*_ and λ_*in*_ are gain factors (or synaptic weights).

In the insect vision system, an ON-OFF channel divides motion information into parallel ON and OFF channels, encoding the brightness increment (ON) and decrement (OFF), respectively. In DFLGMD, an ON-OFF mechanism is realized using half wave rectification and a threshold membrane potential to separate visual processing from the photoreceptor layer for parallel computing:


(2) $$\begin{array}{l} P_{ij}^{ON}\left(t\right)=\gamma_{1}{\left[P_{ij}\left(t\right)-V_{th1}\right]}^{+} \end{array}$$

and


(3) $$\begin{array}{l} P_{ij}^{OFF}\left(t\right)=-\gamma_{2}{\left[P_{ij}\left(t\right)-V_{th2}\right]}^{-}, \end{array}$$

where [.]^+^≡*max*(., 0), [.]^−^≡*min*(., 0); γ_1_ and γ_2_ are gain factors of ON and OFF, respectively; *V*_*th*1_ is the threshold of the ON channel; and *V*_*th*2_ is the threshold of the OFF channel.

### 3.2. Excitatory/inhibition layer

The second layer of the DFLGMD network corresponds to the excitatory and inhibitory neurons in the insect visual system. Excitatory and inhibitory neurons are parallel groups of neurons that can enhance or inhibit the intensity of signals. There is a critical competition between them. If the excitatory neurons “win” the competition, the postsynaptic neurons are successfully activated, causing the corresponding neuronal response. Otherwise, the corresponding postsynaptic neurons cannot respond.

In the proposed neural network, the excitatory neuron *E* is located between the P layer and the S layer, receives the output signal of the ON-OFF channel, and then directly transmits it to the corresponding postsynaptic neuron, i.e.,


(4) $$\begin{array}{l} E_{ij}^{ON}\left(t\right)=P_{ij}^{ON}\left(t\right) \end{array}$$

and


(5) $$\begin{array}{l} E_{ij}^{OFF}\left(t\right)=P_{ij}^{OFF}\left(t\right). \end{array}$$

In the visual nervous system, excitatory neurons directly transmit signals, and inhibitory neurons mainly rely on lateral inhibitory waves to inhibit peripheral neurons. As the inhibition wave produces an information residue in the transmission process that does not disappear immediately, the time at which the signal is received by the postsynaptic neuron is often delayed. Therefore, after passing through a delay unit, the signal produces lateral inhibition of the peripheral neurons.

In DFLGMD, inhibitory neuron *I* is parallel to excitatory neuron *E*, and there is no interaction between signals. The inhibitory neuron *I* first receives the output signal from the ON-OFF channel. The specific model is as follows:


(6) $$\begin{array}{l} D_{t}^{\alpha_{1}}I_{ij}^{ON}\left(t\right)=g_{leak}\left(V_{rest}-I_{ij}^{ON}\left(t\right)\right)+\delta_{ex}P_{ij}^{ON}\left(t\right)\left(E_{ex}-I_{ij}^{ON}\left(t\right)\right), \end{array}$$

where *g*_*leak*_ is the leakage conductance of inhibitory cells; *V*_*rest*_is the resting potential of inhibitory cells; *E*_*ex*_ indicates the excitatory synaptic battery; and δ_*ex*_ is the adjustment coefficient.

In a similar way,


(7) $$\begin{array}{l} D_{t}^{\alpha_{1}}I_{ij}^{OFF}\left(t\right)=g_{leak}\left(V_{rest}-I_{ij}^{OFF}\left(t\right)\right)+\delta_{ex}P_{ij}^{OFF}\left(t\right)\left(E_{ex}-I_{ij}^{OFF}\left(t\right)\right). \end{array}$$

Then,


(8) $$\begin{array}{l} {Î}_{ij}^{ON}\left(t\right)={\left[I_{ij}^{ON}\left(t\right)\right]}^{+} \end{array}$$

(9) $$\begin{array}{l} {Î}_{ij}^{OFF}\left(t\right)={\left[I_{ij}^{OFF}\left(t\right)\right]}^{+}. \end{array}$$

Next, after a time delay, neuron *I* produces a lateral inhibition of the peripheral neurons at different spatial positions. In this work, a suitable spatial inhibition function is selected to describe the inhibition by neurons of the surrounding neurons; this is then convolved with the signal in the delay time to achieve the lateral inhibition function of the DFLGMD network. The specific model is as follows:


(10) $$\begin{array}{l} {Ī}_{ij}^{ON}\left(t\right)={Î}_{ij}^{ON}\left(t\right)⊗G_{1}+\beta_{on}\left[{Î}_{ij}^{ON}\left(t-1\right)⊗G_{2}\right]. \end{array}$$

Similarly, we have


(11) $$\begin{array}{l} {Ī}_{ij}^{OFF}\left(t\right)={Î}_{ij}^{OFF}\left(t\right)⊗G_{1}+\beta_{off}\left[{Î}_{ij}^{OFF}\left(t-1\right)⊗G_{2}\right], \end{array}$$

where β_*on*_ and β_*off*_ are regulatory factors. *G*_1_ and *G*_2_ are the Gaussian kernels, defined as


(12) $$\begin{array}{l} G\left(x,y\right)=\frac{F}{2\pi \sigma^{2}}exp\left(\frac{-\left(x^{2}+y^{2}\right)}{2\sigma }\right). \end{array}$$

**Figure 3:**
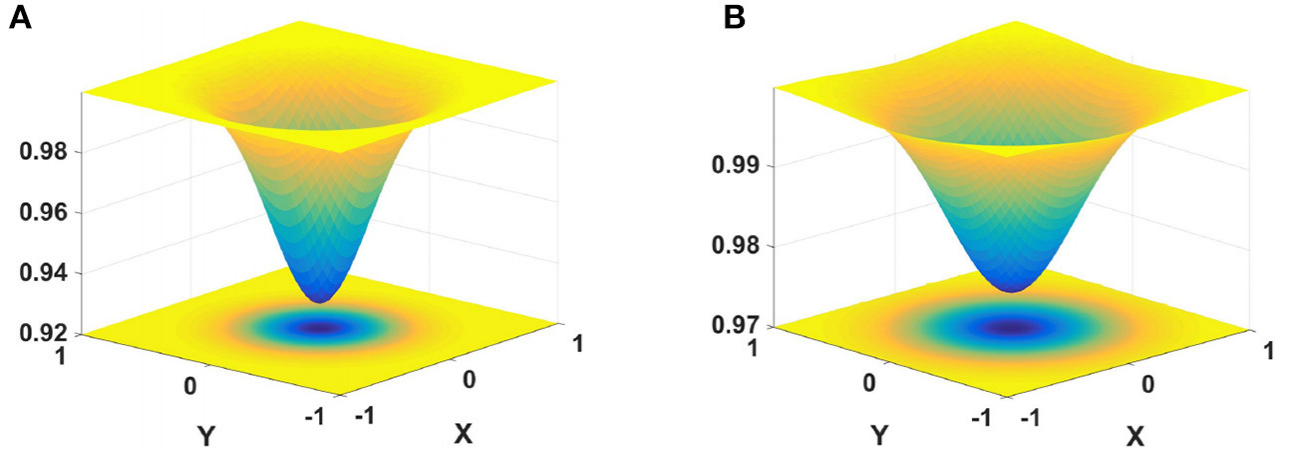
Schematic illustration of cell inhibition. On the left is the nearest neighbors convolution kernel, and on the right is the next-nearest neighbors convolution kernel. **(A)** σ_1_ = 0.3, *F*_1_ = 5, **(B)** σ_2_ = 0.4, *F*_2_ = 1.

The schematic diagram of inhibition kernels *G*_1_ and *G*_2_ is shown in Figure 3. The inhibitory kernels *G*_1_ and *G*_2_ each contain two components, namely, the inhibitory and excitatory regions. The inhibition area of *G*_1_ is small and strong, only inhibiting the adjacent neurons. The inhibition area of *G*_2_ is large, and the surrounding inhibition gradually weakens. *G*_2_ exerts different degrees of inhibition on adjacent neurons according to their spatial positions.

Note that the strength of the inhibition wave decays over time during propagation. Therefore, the proposed neural network has two delay units, and the inhibition strength under different delay units decreases with increasing delay time.

### 3.3. Summing layer

In the visual system of insects, summing neurons are used to express results after critical competition between excitatory and inhibitory neurons. This is closely related to whether or not the membrane potential response of postsynaptic neurons occurs.

In the proposed neural network, a summing layer integrates signals from both excitation and inhibition neurons. First, after the excitation and inhibition of local ON/OFF, both ON and OFF channels have local S-ON and S-OFF summing units, i.e.,


(13) $$\begin{array}{l} D_{t}^{\alpha_{1}}S_{ij}^{ON}(t)=g_{leak}(V_{rest}-S_{ij}^{ON}(t))+\varepsilon_{ex}E_{ij}^{ON}(t)(E_{ex}-S_{ij}^{ON}(t))\\ \;+\varepsilon_{in}{\bar{I}}_{ij}^{ON}(t)(E_{ex}-S_{ij}^{ON}(t)) \end{array}$$

and


(14) $$\begin{array}{l} D_{t}^{\alpha_{1}}S_{ij}^{OFF}(t)=g_{leak}(V_{rest}-S_{ij}^{OFF}(t))+\varepsilon_{ex}E_{ij}^{OFF}(t)(E_{ex}-S_{ij}^{OFF}(t))\\ \;+\varepsilon_{in}{\bar{I}}_{ij}^{OFF}(t)(E_{in}-S_{ij}^{OFF}(t)), \end{array}$$

where *g*_*leak*_ is the leakage conductance of S cells; *V*_*rest*_ indicates the resting potential of S cells; *E*_*ex*_ and *E*_*in*_ indicate excitatory and inhibitory synaptic cells, respectively; and ε_*ex*_ and ε_*in*_ are the weights of excitatory and inhibitory synapses, respectively.

Then,


(15) $$\begin{array}{l} {Ŝ}_{ij}^{ON}\left(t\right)={\left[S_{ij}^{ON}\left(t\right)\right]}^{+} \end{array}$$

(16) $$\begin{array}{l} {Ŝ}_{ij}^{OFF}\left(t\right)={\left[S_{ij}^{OFF}\left(t\right)\right]}^{+}. \end{array}$$

The local signals from the ON-OFF channel interact with each other. After a superlinear operation, the S summing neuron integrates local signals to obtain the final global signal response. The calculation obeys a superlinear rule, as follows:


(17) $$\begin{array}{l} S_{ij}\left(t\right)=\mu_{1}\cdot {Ŝ}_{ij}^{ON}\left(t\right)+\mu_{2}\cdot {Ŝ}_{ij}^{OFF}\left(t\right)+\mu_{3}\cdot {Ŝ}_{ij}^{ON}\left(t\right)\cdot {Ŝ}_{ij}^{OFF}\left(t\right), \end{array}$$

where {μ_1_, μ_2_, μ_3_} denotes the combination of term coefficients that allows the S unit to represent different “balances” between local polarity excitations and mediate influences via ON and OFF contrast.

### 3.4. Direction layer

In the insect visual system, there is a special visual neuron that has a preference for specific directional motion information, called the directionally selective neuron. It can quickly and reliably extract visual motion information in different directions and shows a strong response to object motion in the preferred direction. This is helpful in identifying the direction of an object's motion and is very important for sensing the collision threat of moving objects.

In the DFLGMD neural network, we introduce a directional selectivity mechanism to extract the directional information of moving objects. This mechanism simulates the directionally selective neurons of insects, in that it also shows strong responses to signals in the preferred direction but weak or no response to motion signals in the zero direction (non-preferred direction).

In the fourth layer of the DFLGMD network, directionally selective neurons receive motion information after S-layer integration. Given a preferred direction θ, the direction information of position (*i, j*) on the θ is defined as:


(18) $$\begin{array}{l} D_{ij}\left(t,\theta \right)=S_{ij}\left(t\right)\times S_{x\left(i,\theta \right)y\left(j,\theta \right)}\left(t-2\right)-S_{ij}\left(t-1\right)\times S_{x\left(i,\theta \right)y\left(j,\theta \right)}\left(t-1\right), \end{array}$$

where


(19) $$\begin{array}{l} \begin{array}{c} x\left(i,\theta \right)=i+mcos\theta ,\\ y\left(j,\theta \right)=j+msin\theta , \end{array} \end{array}$$

and *m* is a constant, $\theta \in \left\{0,\frac{\pi }{4},\frac{\pi }{2},\frac{3\pi }{4},\pi ,\frac{5\pi }{4},\frac{3\pi }{2},\frac{7\pi }{4}\right\}$.

**Figure 4:**
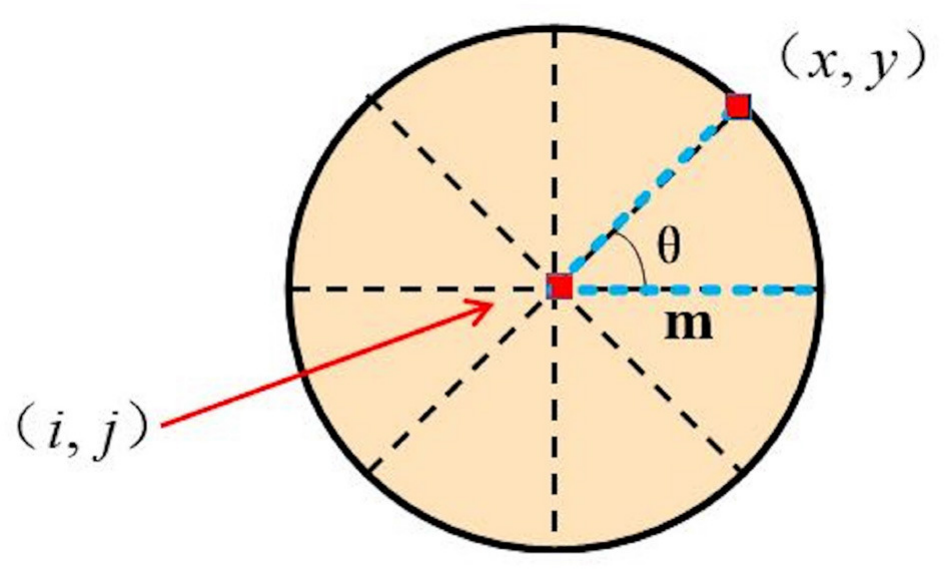
Schematic illustration of relative position between (*i, j*) and (*x, y*). *m* is the distance between (*i, j*) and (*x, y*) while θ is the angle between the directions.

A schematic illustration of the relative position between (*i, j*) and (*x, y*) is presented in Figure 4. For a given position (*i, j*), we can choose a series of (*x, y*) corresponding to different directions θ. Thus, a series of correlation outputs *D*_*ij*_(*t*, θ) with different preferred motion directions θ can be defined. For a given direction θ_0_, *D*_*ij*_(*t*, θ_0_) gives the strongest output in response to object motion oriented along direction θ_0_, with weak or no output in response to motion oriented along other directions. That is, *D*_*ij*_(*t*, θ_0_) shows directional selectivity.

### 3.5. LGMD layer

In the insect visual system, LGMDs, a class of collision-sensitive neurons, are very sensitive to the movement of looming objects but show little response to the movement of distant objects.

In the proposed neural network, after a series of presynaptic steps to process visual information, the LGMD neuron receives local information from the D layer and integrates it as follows:


(20) $$\begin{array}{l} R_{1}\left(t,\theta \right)=\overset{n}{\underset{i,j}{\sum }}D_{ij}\left(t,\theta \right), \end{array}$$

where *n* is the number of rows or columns of the neuron layer.

Further, the response activity of LGMD neurons with directional selectivity in direction θ is


(21) $$D_{t}^{\alpha_{1}}L_{1}(t,\theta )=g_{leak}(V_{rest}-L_{1}(t,\theta ))+{\text{ξ}}_{ex}R_{1}(t,\theta )(E_{ex}-L_{1}(t,\theta )),$$

where $\xi_{ex}=5\times 28^{2}/n^{2}$ is a gain factor, and *n* is the number of rows or columns of the neuronal layer. Finally,


(22) $$\begin{array}{l} \hat{L_{1}}\left(t,\theta \right)={\left[L_{1}\left(t,\theta \right)\right]}^{+}. \end{array}$$

Here, $\hat{L_{1}}\left(t,\theta \right)$ has the strongest response to objects moving in the θ direction and weak or no response to objects moving in other directions. That is, $\hat{L_{1}}\left(t,\theta \right)$ represents the directionally selective response activity of the neural network.

### 3.6. Motion direction estimation

In this subsection, we introduce a method for estimating the motion direction of objects based on the direction information obtained by the DFLGMD visual neural network. This method utilizes the model's output to estimate the direction of moving objects along both the horizontal and vertical axes.

First, we used directionally selective neurons to obtain local directional information moving in horizontal and vertical directions, that is,


(23) $$\begin{array}{l} D_{ij}\left(t,0\right)=S_{ij}\left(t\right)\times S_{x\left(i,0\right)y\left(j,0\right)}\left(t-2\right)-S_{ij}\left(t-1\right)\times S_{x\left(i,0\right)y\left(j,0\right)}\left(t-1\right) \end{array}$$

and


(24) $$\begin{array}{l} \begin{array}{c} D_{ij}\left(t,\frac{\pi }{2}\right)=S_{ij}\left(t\right)\times S_{x\left(i,\frac{\pi }{2}\right)y\left(j,\frac{\pi }{2}\right)}\left(t-2\right)-S_{ij}\left(t-1\right)\\ \times S_{x\left(i,\frac{\pi }{2}\right)y\left(j,\frac{\pi }{2}\right)}\left(t-1\right). \end{array} \end{array}$$

Then, the arc tangent function is used to obtain the local motion direction angle of the object at time *t*:


(25) $$\begin{array}{l} E_{ij}\left(t\right)=arctan\left(D_{ij}\left(t,\frac{\pi }{2}\right),D_{ij}\left(t,0\right)\right). \end{array}$$

Finally, the motion direction angle of the object can be obtained by integrating all local direction information; that is,


(26) $$\begin{array}{l} MD\left(t\right)=\Theta \left(E_{ij}\left(t\right),i=1,2,.....n;j=1,2,.....n\right), \end{array}$$

where *MD*(*t*) denotes the motion direction of the object at time *t*, Θ is the mode operation, and *n* is the number of rows or columns of matrix *E*.

## 4. Experiments and discussion

In this section, we test the performance of the proposed DFLGMD visual neural network. The experiments presented here include three experimental scenes, simulated scenarios, real-world scenarios, and real complex scenarios. In the following subsections, we introduce the system parameters and experimental methods used for various scenarios and present the experimental results with some discussion.

### 4.1. Parameters of the system

Parameters of the proposed DFLGMD are given in Table 1. The proposed neural network has a large number of parameters, and there is no learning method for parameter setting at present. Therefore, these parameters were tuned manually based on empirical experience. When the neural network system is applied to cluttered and dynamic scenes, some parameters need to be fine tuned. Therefore, ranges for some parameters are given in Table 1. Without special emphasis, the parameters do not exceed the given range.

**Table 1:** Setting parameters of the proposed DFLGMD model.

**Parameter**	**Description**	**Value**
α_1_	The order of fractional-order differential operators	0.4
*g*_*leak*_	The leakage conductance which the cell	[25, 50]
*V*_*rest*_	The resting potential which the cell	[−0.001, 0]
*V*_*th*1_	A threshold membrane potential	0.0005
*V*_*th*2_	A threshold membrane potential	0.0005
*E*_*in*_	The inhibitory synaptic batteries	[−1, −0.3]
*E*_*ex*_	The excitatory synaptic batteries	1.0
λ_*ex*_	A gain factors	1.2
λ_*in*_	A gain factors	1.2
γ1	A gain factors	150
γ2	A gain factors	150
ε_*ex*_	The weights of excitatory synapses	1.0
ε_*in*_	The weights of inhibitory synapses	100
δ_*ex*_	The adjustment coefficient	1.5
β_*on*_	A regulatory factors	[1.0, 1.2]
β_*off*_	A regulatory factors	[1.0, 1.2]
*m*	A distance constant	1.0
*F*_1_	The coefficient of Gaussian kernel G1	5
*F*_2_	The coefficient of Gaussian kernel G2	1
σ_1_	The variance of Gaussian kernel G1	0.3
σ_2_	The variance of Gaussian kernel G2	0.4
ξ_*ex*_	A gain factors	5 × 128^2^/*n*^2^
{μ_1_, μ_2_, μ_3_}	The term coefficients	{1, 1, 0}

The proposed neural network was written in MATLAB 2020a (MathWorks, Inc., Natick, MA). The experimental environment was Microsoft Windows 10 operating system, with an Inter(R) Core(TM) i5-3470S CPU @2.90 GHz processor and 8 G memory.

### 4.2. Simulated scenarios

In order to test the basic performance of the algorithm proposed in this paper, we selected eight groups of simulation videos showing objects moving in different directions for testing purposes. The videos included two groups of object movements in the depth direction (including looming motion and faraway motion) and six groups of object movements in other directions (left, right, up, down, left upper corner, and right lower corner).

**Figure 5:**
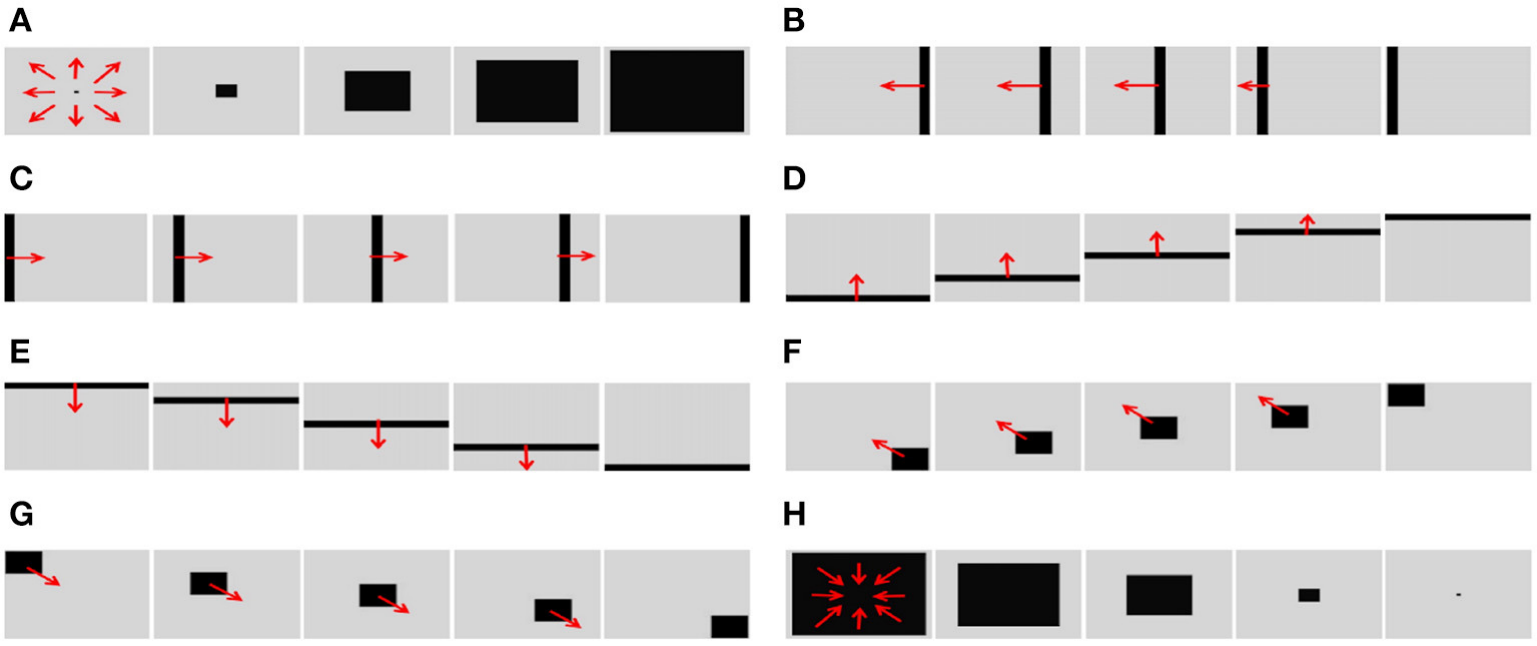
Example frames of videos. The video includes two groups **(A, H)** of object movements in the depth direction (including looming motion and faraway motion) and six groups **(B–G)** of object movements in other directions (left, right, up, down, left upper corner, and right lower corner).

**Figure 6:**
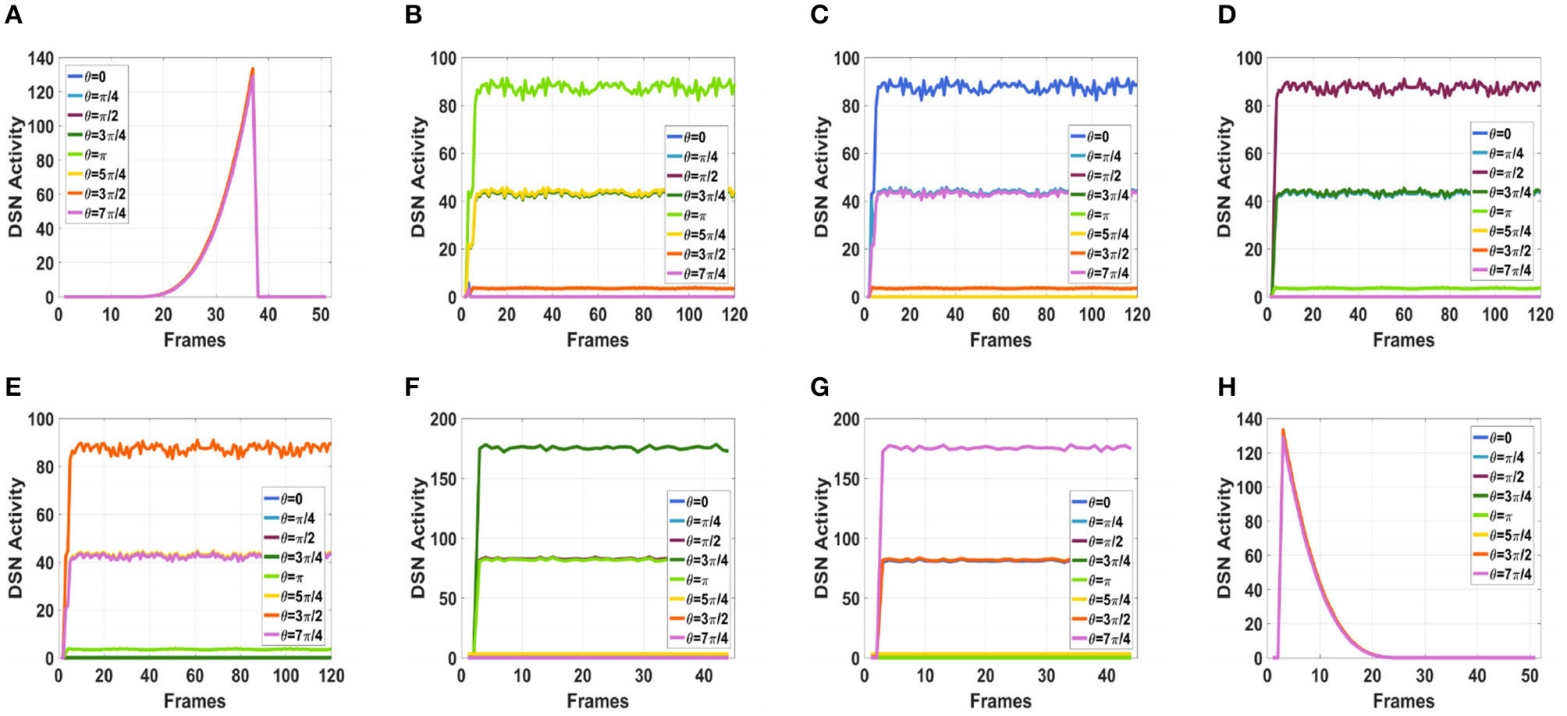
Simulation results for the directionally selective fractional-order LGMD collision sensing visual neural network. **(A)** Object looming motion, **(B)** object translating leftwards, **(C)** object translating rightwards, **(D)** object translating upwards, **(E)** object translating upwards, **(F)** object left upper corner motion, **(G)** object right lower corner motion, and **(H)** object faraway motion.

**Figure 7:**
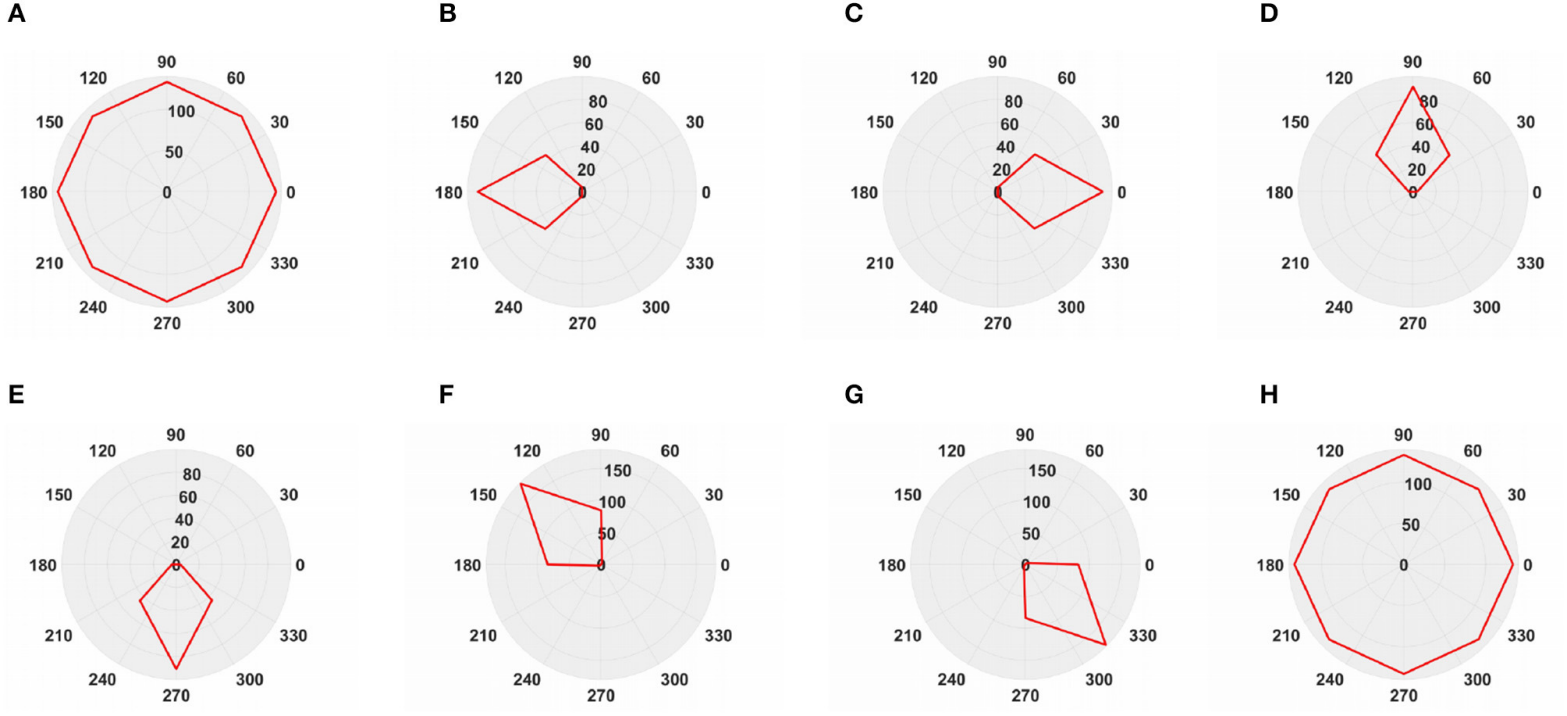
The output direction of directionally selective fractional-order LGMD collision sensing visual neural network. **(A)** Object looming motion, **(B)** object translating leftwards, **(C)** object translating rightwards, **(D)** object translating upwards, **(E)** object translating upwards, **(F)** object left upper corner motion, **(G)** object right lower corner motion, and **(H)** object faraway motion.

To simulate the movement of objects in the depth direction, we used the diffusion movement of small black blocks to represent objects moving forward at a constant speed in the depth direction. During the movement, the positions of the small black blocks remained unchanged, and their size gradually increased. When the small black blocks filled the entire field of vision, their size did not change again; that is, the field of vision did not change. This moment was defined as the collision time. Video A simulates the looming motion of an object, and video H simulates the far motion of the object. The motion direction in video H was exactly opposite to that of video A. We used the contraction of the small black blocks to simulate uniform motion toward the rear in the depth direction. The smaller the size of the small black block, the farther away the simulated object. In the other six groups of videos (B–G), we used the movements of bars and small black blocks in different directions to simulate the movements of objects. During movement, the sizes of the grid bar and the small black blocks remained unchanged; only the direction of movement changed, and no collision occurred. The motion directions in videos B–G were from right to left, from left to right, from bottom to top, from top to bottom, from 135 degrees up, and from 315 degrees down. The experimental sampling frequency was 33.3 ms, and the pixel size was 128 × 128. Some frames from videos A–H are shown in Figures 5A–H. The corresponding results are shown in Figures 6, 7, where Figure 6 shows the output activity of the fractional-order collision perception visual neural network with direction selectivity, referred to as the direction-selective network, and Figure 7 shows the object motion direction perceived by the network.

**Figure 8:**
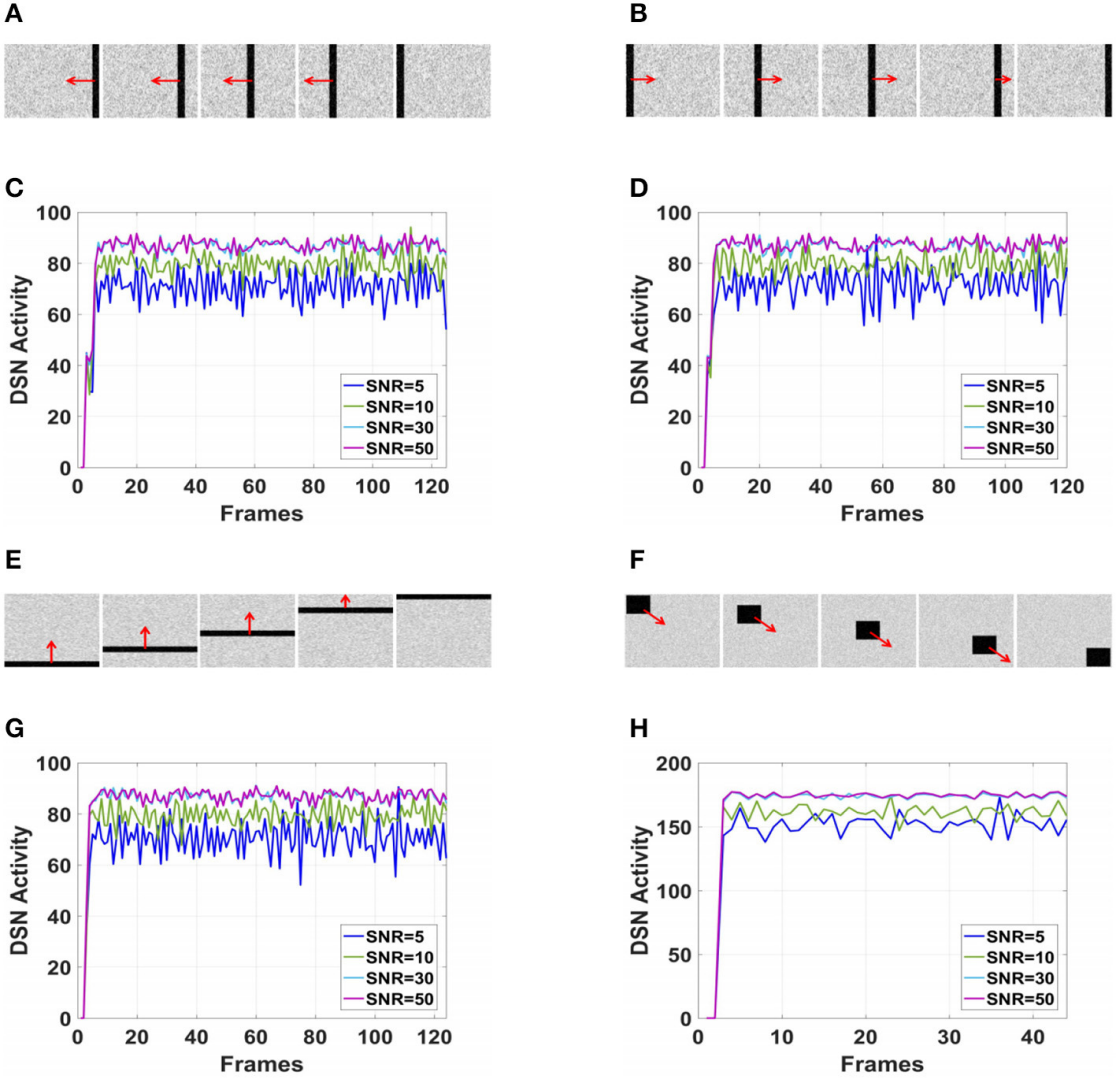
The output of proposed visual neural networks in real motion directions under different signal-to-noise ratios. **(A)** Object translating leftwards. **(B)** Object translating rightwards. **(C)** θ = π. **(D)** θ = 0. **(E)** Object translating upwards. **(F)** Object right lower corner motion. **(G)** θ = π/2. **(H)** θ=7π/4.

Considering the robustness of the proposed neural network to noise, this paper added Gaussian noise with different signal-to-noise ratios (SNRs) to the simulated videos during the testing process. The Gaussian noise follows the normal distribution *N*(0, 1), and the SNRs are set to 5, 10, 30, and 50. During the experiment, the motion direction was fixed, and the network outputs under different SNRs were compared. The frames and results corresponding to the videos are shown in Figure 8.

As shown in Figures 6A–H, DFLGMD accurately perceived the collision threat but did not respond to translation movement in any direction. Specifically, the network output corresponding to looming video A reached its peak at frame 37, consistent with the real collision time in video A. Videos B–G show translational motions in different directions. As shown in Figures 6B–G, the neural activity output by the network tended to be flat, with no obvious peak. DFLGMD accurately perceived the looming motion in the video and showed good collision perception ability. Figures 7A–H shows the object motion direction perceived by the DFLGMD neural network. The motion direction of the object corresponding to videos B–G was consistent with the experimental results. The neuron showed high response activity to the motion in the preferred direction but no or little response to motions in other directions. In videos A and H, small black blocks diffused or contracted evenly in all directions when objects were looming or far away, and motion could be perceived in each direction; as shown in Figures 7A, H, neurons had responses in each direction with similar response activity. Figure 8 shows the network outputs of DFLGMD under different signal-to-noise ratios. It can be observed that as the SNR decreases, the output activity of the algorithm decreases, but the overall change is not significant, indicating that the algorithm has good robustness. In summary, these results show that DFLGMD achieves the two basic functions of sensing a collision and recognizing object motion direction, owing to the basic characteristics of LGMDs and directionally selective neurons, and the network performance was good.

### 4.3. Real-world scenarios

**Figure 9:**
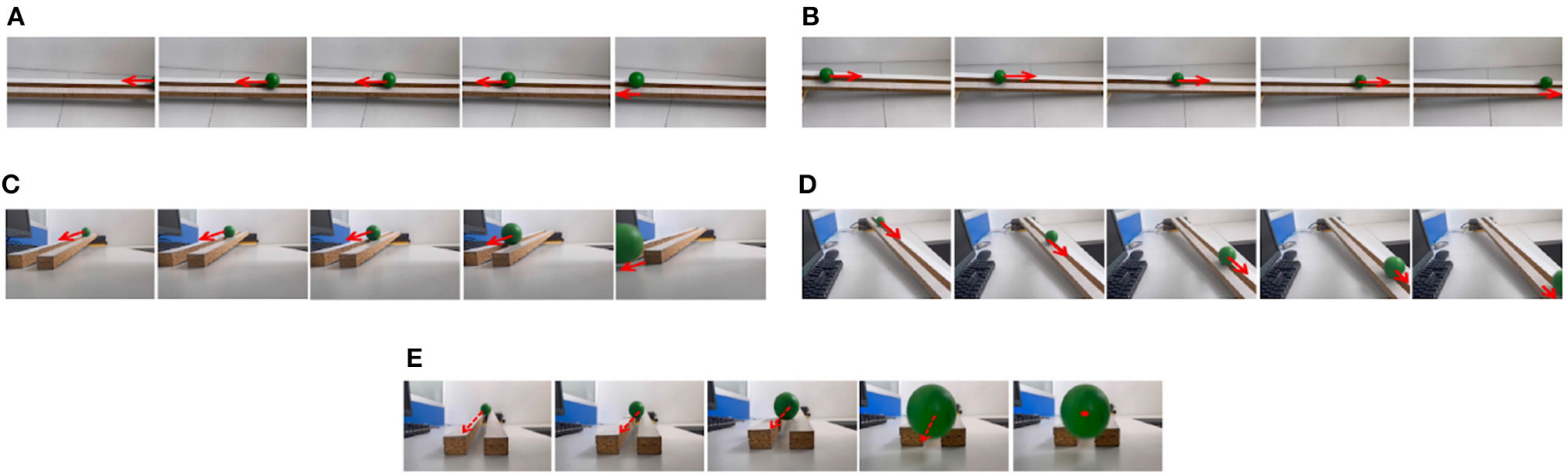
Example frames of ball motion videos. **(A)** The ball moves to the left. **(B)** The ball moves to the right. **(C)** The ball moves downwards to the left. **(D)** The ball moves downwards to the right. **(E)** The ball is looming moving toward the center.

**Figure 10:**
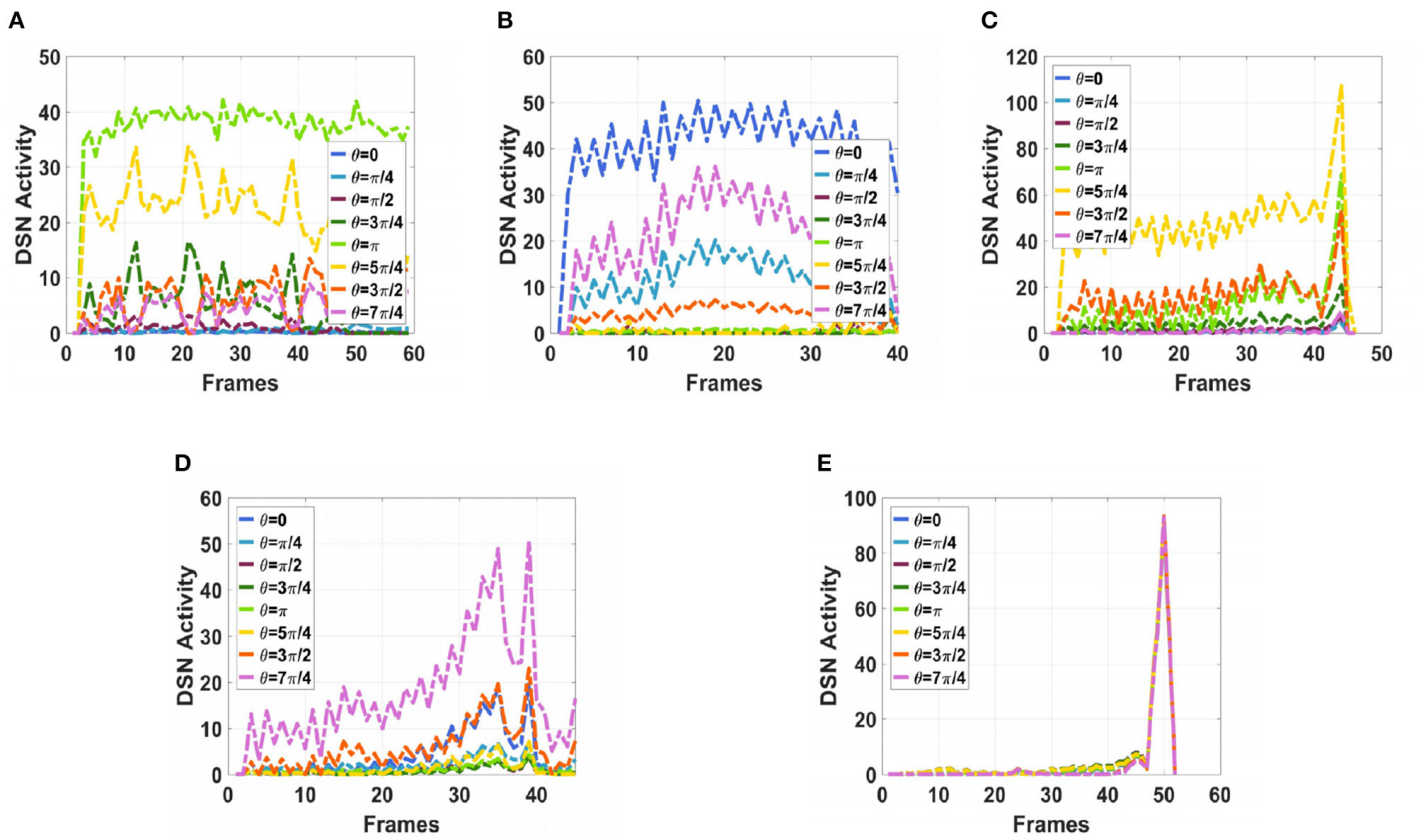
The output activity of the directionally selective fractional-order LGMD collision sensing visual neural network. **(A)** The ball moves to the left. **(B)** The ball moves to the right. **(C)** The ball moves downwards to the left. **(D)** The ball moves downwards to the right. **(E)** The ball is looming moving toward the center.

**Figure 11:**
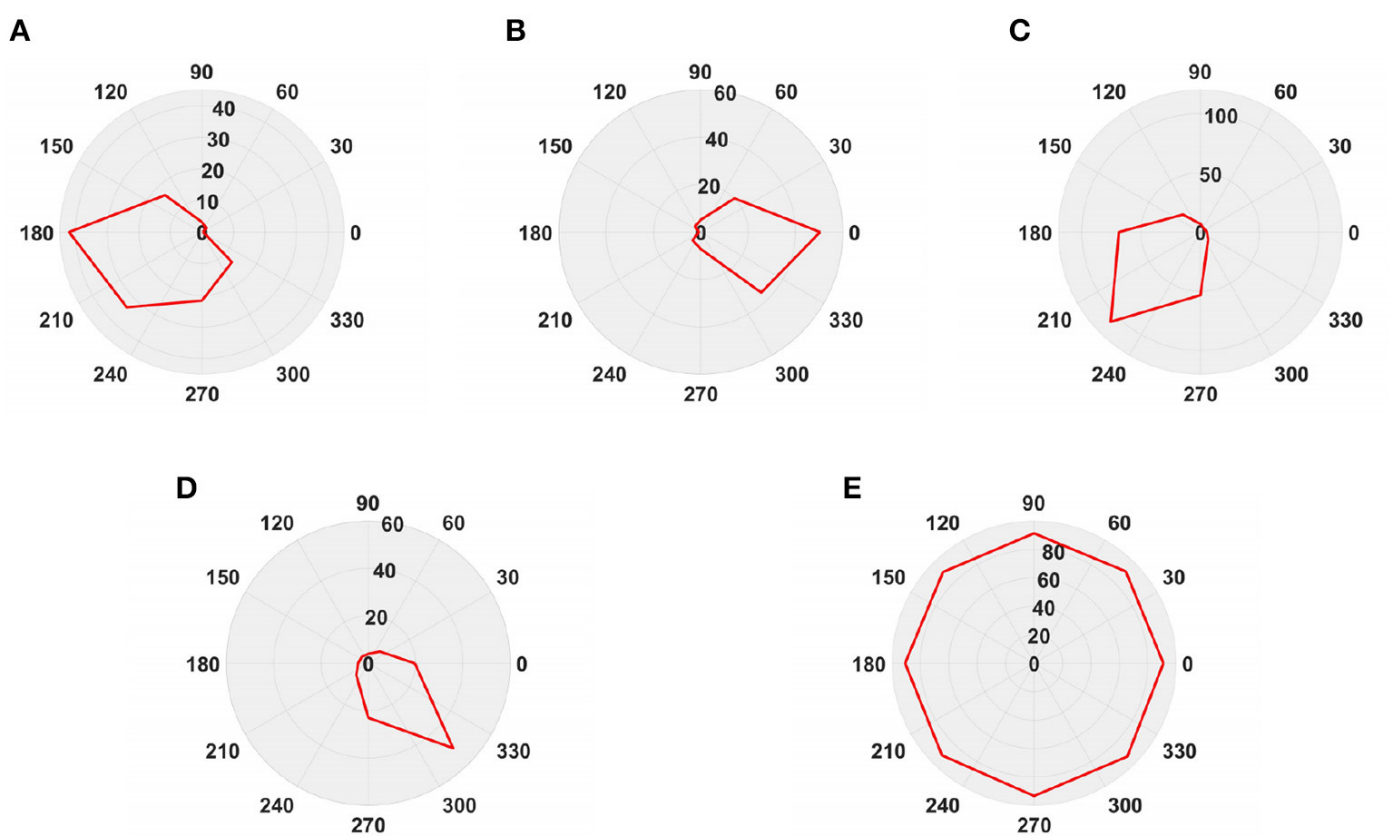
The output direction of the directionally selective fractional-order LGMD collision sensing visual neural network. **(A)** The ball moves to the left. **(B)** The ball moves to the right. **(C)** The ball moves downwards to the left. **(D)** The ball moves downwards to the right. **(E)** The ball is looming moving toward the center.

**Figure 12:**
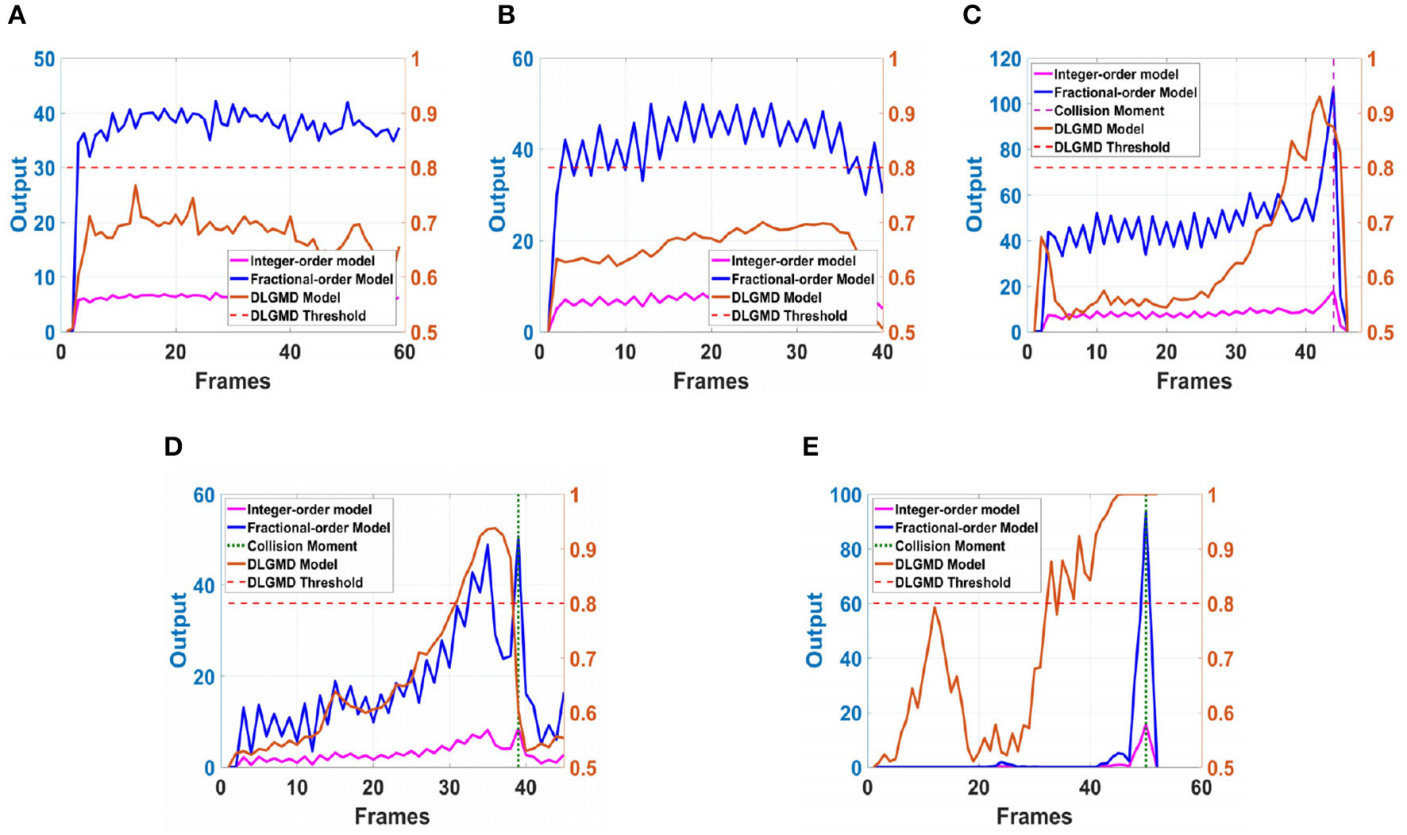
The neural network output of different models in ball motion video. **(A)** The ball moves to the left. **(B)** The ball moves to the right. **(C)** The ball moves downwards to the left. **(D)** The ball moves downwards to the right. **(E)** The ball is looming moving towards the center.

Next, in order to better test the performance of the DFLGMD neural network, we used real physical scenes for experiments. First, we built a simple experimental platform in the laboratory. During the experiments, we specified the direction of motion in advance. The ball moved in the specified direction. The closer the ball to the camera during the movement, the larger it appeared in the field of vision. There were five videos in total, and the ball moved in five different directions, as shown in Figures 9A–E. The experimental sampling frequency was 33.3 ms, and the pixel size was 128 × 128. The experimental results are shown in Figures 10, 11. Figure 10 shows the network output of the LGMD neural network with direction selectivity for the five videos in turn, and Figure 11 shows the motion direction perceived by the network model proposed in this paper. Then, to verify the network performance of DFLGMD, we designed comparative experiments. In the experiment, we compared the neural network proposed in this paper with the integer-order LGMD neural network and the representative DLGMD neural network (Yue and Rind, 2013). The structure of the integer-order LGMD network is the same as the neural network proposed in this paper, with a differential operator order of 1. The experimental results are shown in Figure 12.

As shown in Figures 10A, 11A, the output activity of the network was relatively flat during the whole movement process of the small ball, with no prominent peak. The network output activity was highest when θ = π, indicating that the small ball moved from right to left without collision threat. Similarly, in Figure 11B, the network output activity was highest when θ = 0; that is, the ball moved from left to right, again without collision threat. Figures 11C, D shows the case where the small ball was looming in the video. In Figure 10C, the peak was reached in frame 43, whereas in Figure 10D it was reached in frame 39, consistent with the real collision time. Figures 11C, D show the motion direction of the small ball in the looming process. In Figure 11E, the up-down inclination angle of the wooden strip was set to be small, and the ball approached almost uniformly from the right center. As shown in Figure 10E, the peak was reached at 50 frames, and the collision occurred. This uniform approaching from the right center can be seen as a diffusion movement to the surroundings. As shown in Figure 11E, the response was the same in all directions. From Figure 12, it can be seen that the detection of direction and collision by the DLGMD neural network depends on the size of the threshold. When the output is higher than the threshold, the neuron responds, otherwise it does not respond. The time when the collision occurred in the looming video is also marked in Figure 12. It can be seen that the DFLGMD can accurately detect the occurrence of collisions compared to the DLGMD, which is due to the two different response modes of the neurons mentioned earlier. At the same time, it can be seen that the output of the fractional-order model is significantly higher than that of the integer-order model, that is, the fractional-order model characterizes the neuron response more closely and reliably.

**Figure 13:**
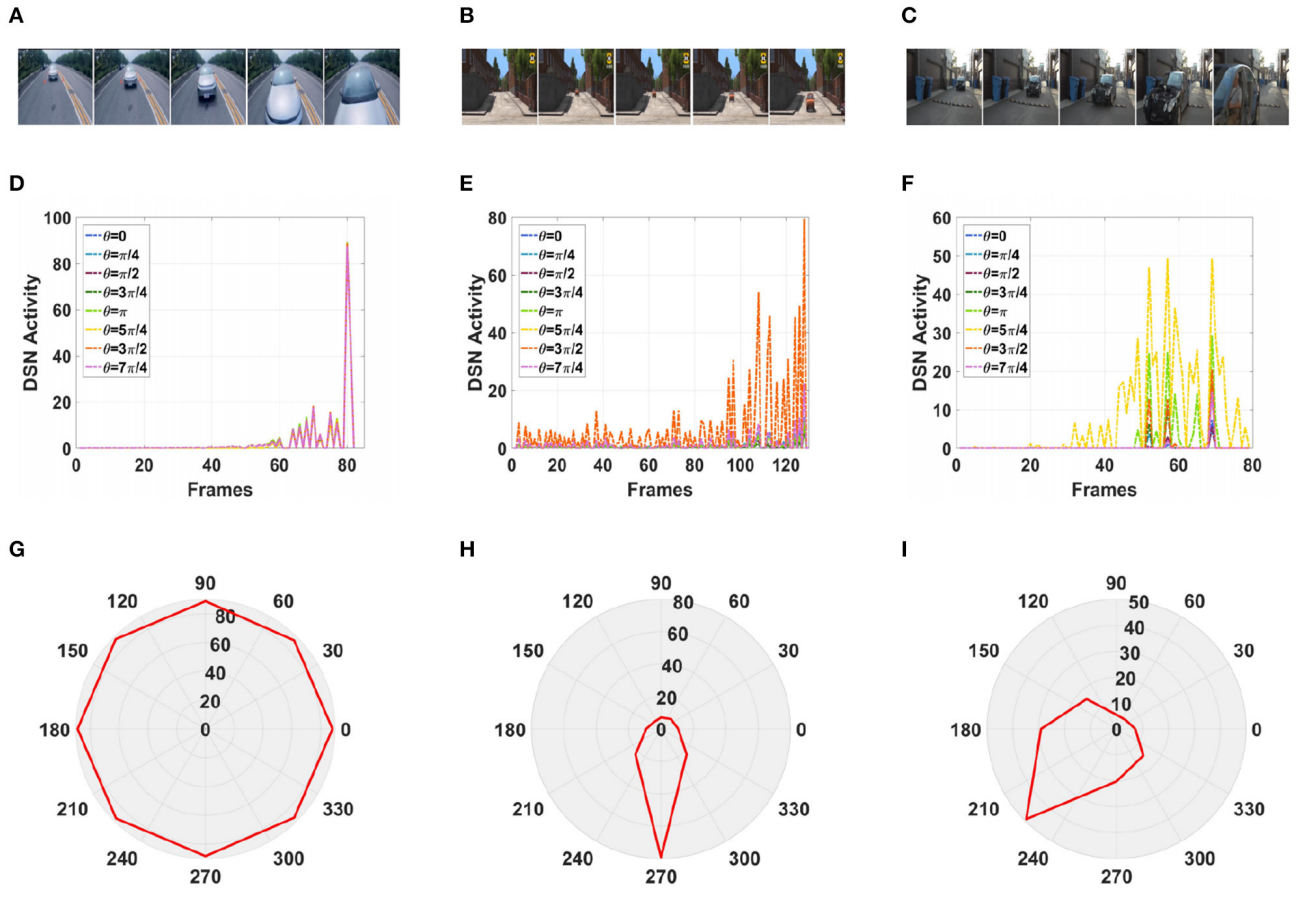
Simulation results for the directionally selective fractional-order LGMD collision sensing visual neural network in real complex videos. **(A–C)** Are the example frames of videos. **(D–F)** Are the output activity of the neural network. **(G–I)** Are the output direction of the neural network.

**Figure 14:**
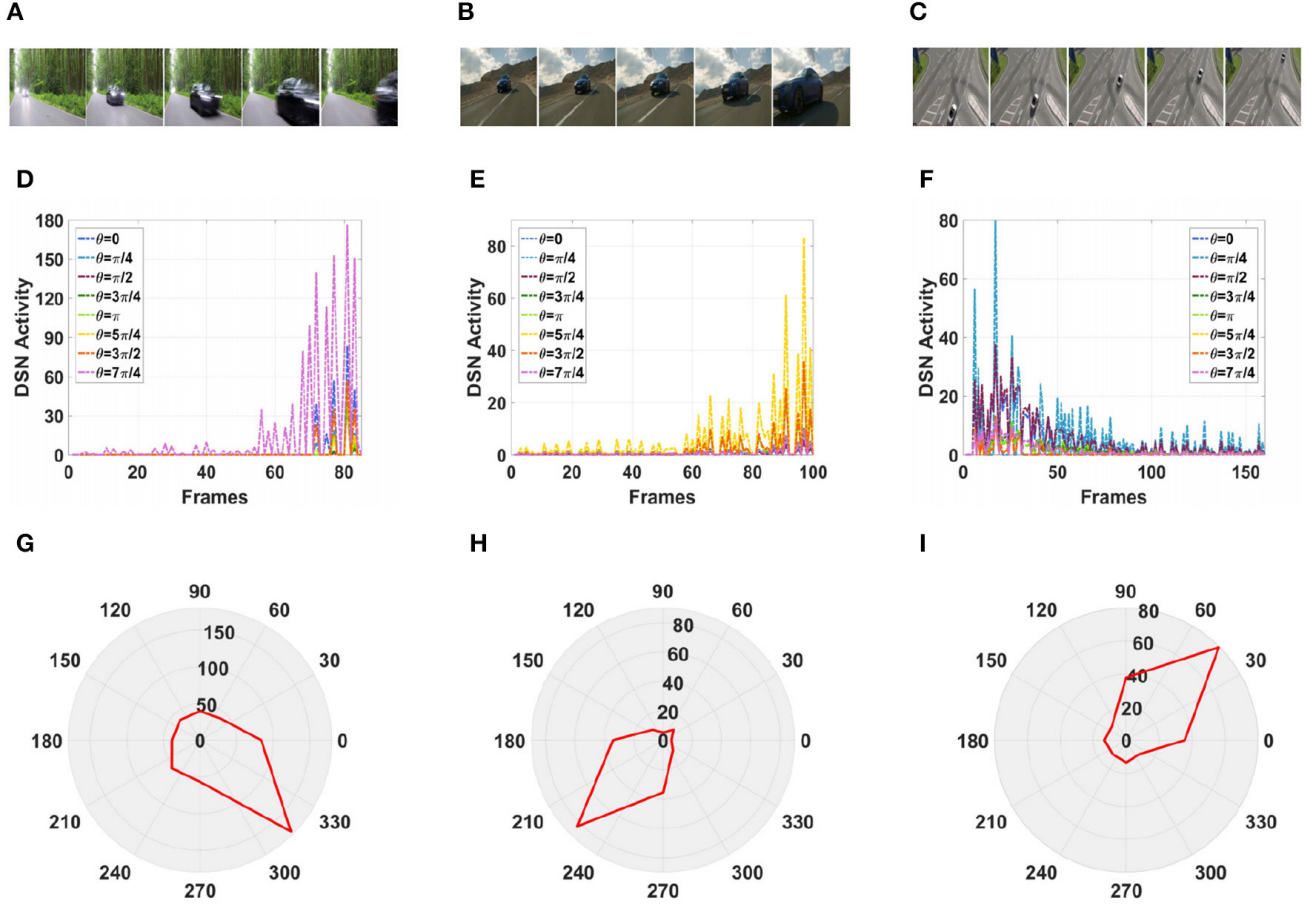
Simulation results for the directionally selective fractional-order LGMD collision sensing visual neural network in real complex videos. **(A–C)** Are the example frames of videos. **(D–F)** Are the output activity of the neural network. **(G–I)** Are the output direction of the neural network.

Furthermore, in a real-life scenario, the road conditions are changeable and the video background is complex and can change at any time. Therefore, we tested our model using a complex road scene in a real physical environment. We selected six real road videos in which the target vehicle moved in different directions. The vehicle in video 1 was approaching from the front, and the vehicle in video 2 was approaching from the front on a downhill road. The vehicles in videos 3, 4, and 5 were approaching from the left and right, respectively. The vehicle in video 6 was moving away from the road and gradually disappeared from the field of vision. The experimental sampling frequency was 33.3 milliseconds, and the pixel size was 128 × 128. The partial frames extracted from each video and the corresponding experimental results are shown in Figures 13, 14.

As shown in Figures 13D, G, the video reached its peak at frame 80, so there was a collision. The collision event was at the moment when the peak occurred, and the car was approaching from the center. As shown in Figures 13E, H, the vehicle moved downward as it was approaching, and there was a collision. In the three groups of figures (Figures 13C, 14A, B), the vehicle is shown to be approaching, and the motion direction is shown in Figures 14I, G, H. In Figure 14C, the vehicle is driving far away, and its motion direction is shown in Figure 14I.

The above results demonstrate that DFLGMD can not only perceive a collision threat but also obtain the direction of motion of the collision object, leading to more comprehensive network awareness performance compared with other methods.

## 5. Further discussions

In general, the proposed neural network model has successfully achieved its main objective, which is to generate LGMD and direction-selective neurons that respond to approaching objects and their motion direction. The system can sensing the collision threat of looming objects in real-world scenarios while capturing the motion direction of the objects. This means that it can provide reliable guidance for various collision avoidance path planning, which will play a positive role in future work.

In the visual system of insects, many neurons work together. An interesting possibility in our model is the role of feed forward inhibition (FFI) neurons and their incoming information. FFI neurons provide forward inhibition to LGMD by selectively inhibiting high-speed or suddenly appearing looming objects, allowing the early small signals produced during the object approaching process to be distinguished. Since the deep representation of FFI neurons is relatively new and the exact nature of their incoming fibers is not clear, they were not modeled in this paper. Currently, physiological research on FFI is only at a preliminary stage. Recently, Olson et al. (2021) modeled FFI based on its anatomical structure and electrophysiological characteristics, but this model was only used to perceive the collision threat of looming objects. Integrating the information of FFI with LGMD and direction-selective neurons may help improve the network performance of DFLGMD. In the future, we need to consider the collaborative work of these neurons.

We also noticed another interesting possibility regarding the propagation of lateral inhibition. Lateral inhibition has two modes of propagation: the first is delayed propagation, which is due to the inherent characteristics of synaptic communication and results in the information being delayed. The second is diffusive propagation, which occurs due to the difference in dynamics between excitatory and inhibitory receptors, causing lateral inhibition to spread to adjacent principal cells. Due to the motion of particles and complex dynamics involved in diffusion-based propagation, most current models are based on delayed propagation for simplicity's sake. However, given the unique nature of diffusive inhibition, we note that the diffusive propagation of inhibition may have interesting computational properties, which could be a potential improvement in modeling.

A significant limitation of the model proposed in this paper is the parameter setting of the system. The DFLGMD system contains many parameters that are crucial to the system. However, determining accurate parameters is often difficult. In the experiments conducted in this article, most parameters were manually adjusted based on experience, which may result in differences in accuracy compared to real biological neurons. In addition, due to the complexity and uncertainty of real-time road experiment, the model proposed in this paper has not completed the test of real-time road system. However, these limitations have not changed the basic functionality of the model. On the contrary, they have provided us with directions for further work. We will consider introducing deep learning to adjust system parameters and designing high-performance real-time systems to improve the system's robustness in complex dynamic scenarios.

## 6. Conclusion

In this paper, we propose a DFLGMD collision-sensing visual neural network. In the proposed neural network, a new association mechanism is used to obtain direction information for a moving object. Then, an ON/OFF visual channel is used to encode the increase or decrease in brightness, respectively, resulting in selectivity of the system for looming bright or dark objects. Finally, a directionally selective neuron is fused with the fractional-order collision-perception visual neural network, enabling the system to obtain the motion direction of the collision object while sensing the collision threat, as well as making the correct collision avoidance decision. In this work, the DFLGMD visual neural network was systematically studied and tested in different environments. Experimental results show that the DFLGMD visual neural network produces reliable response spikes in response to looming objects and can also accurately obtain the movement direction of approaching objects, with more comprehensive network performance compared with other methods. In future work, we plan to investigate the computational properties of diffusion propagation and model it using an inhibitory diffusion propagation method. Additionally, we will attempt to introduce FFI neurons and study the collaborative relationship between FFI, LGMD, and direction-selective neurons to optimize the performance of the neural network.

## Data availability statement

The original contributions presented in the study are included in the article/supplementary material, further inquiries can be directed to the corresponding author.

## Author contributions

YW designed the network system, performed the experiment, and wrote the first draft of the manuscript. HL contributed to the conception and design of the study and wrote sections of the manuscript. YZ participated in some experiments. JP contributed to the conception and design of the study. All authors contributed to manuscript revision and read and approved the submitted version ideas of the project and review.
